# Divergent Macroparasite Infections in Parapatric Swiss Lake-Stream Pairs of Threespine Stickleback (*Gasterosteus aculeatus*)

**DOI:** 10.1371/journal.pone.0130579

**Published:** 2015-06-18

**Authors:** Anssi Karvonen, Kay Lucek, David A. Marques, Ole Seehausen

**Affiliations:** 1 University of Jyväskylä, Department of Biological and Environmental Science, FI-40014 University of Jyväskylä, Jyväskylä, Finland; 2 Eawag, Centre of Ecology, Evolution and Biogeochemistry, Department of Fish Ecology and Evolution, Kastanienbaum, Switzerland; 3 Division of Aquatic Ecology & Macroevolution, Institute of Ecology and Evolution, University of Bern, Bern, Switzerland; University of Calgary, CANADA

## Abstract

Spatial heterogeneity in diversity and intensity of parasitism is a typical feature of most host-parasite interactions, but understanding of the evolutionary implications of such variation is limited. One possible outcome of infection heterogeneities is parasite-mediated divergent selection between host populations, ecotypes or species which may facilitate the process of ecological speciation. However, very few studies have described infections in population-pairs along the speciation continuum from low to moderate or high degree of genetic differentiation that would address the possibility of parasite-mediated divergent selection in the early stages of the speciation process. Here we provide an example of divergent parasitism in freshwater fish ecotypes by examining macroparasite infections in threespine stickleback (*Gasterosteus aculeatus*) of four Swiss lake systems each harbouring parapatric lake-stream ecotype pairs. We demonstrate significant differences in infections within and between the pairs that are driven particularly by the parasite taxa transmitted to fish from benthic invertebrates. The magnitude of the differences tended to correlate positively with the extent of neutral genetic differentiation between the parapatric lake and stream populations of stickleback, whereas no such correlation was found among allopatric populations from similar or contrasting habitats. This suggests that genetic differentiation is unrelated to the magnitude of parasite infection contrasts when gene flow is constrained by geographical barriers while in the absence of physical barriers, genetic differentiation and the magnitude of differences in infections tend to be positively correlated.

## Introduction

Parasitism is an important selective factor in natural host populations, but the diversity and intensity of infections is often spatially heterogeneous. Such heterogeneity may emerge as a consequence of spatial aggregation of infected hosts that release parasite infective stages (i.e. infection hot spots) or because of ecological specialization, i.e. differences in preference of host individuals and populations towards habitats or diets with variable risk of infection [1–6]. Despite the widespread nature of this phenomenon, evolutionary implications of spatially variable infection risk are poorly understood. One possible outcome is parasite-mediated divergent selection where host populations that occupy different habitats and experience different regimes of parasitism may become genetically differentiated and/or reproductively isolated through direct natural selection, pleiotropic effects on mate choice of divergent evolution in the genes of the immune system, or parasite-mediated divergent sexual selection [7–10]. Recently, studies describing differentiated parasite infections among ecologically specialized individuals, well-defined ecotypes, morphs or closely related species of freshwater fishes have begun to accumulate [1, 11–20]. This shows that some of the key conditions for parasite-mediated divergent selection are often met in natural populations. For such selection to facilitate ecological speciation, the differences in infections between ecotypes should show consistency through time and exceed the variation among individuals within populations [1, 3, 21]. Yet, there have been very few comparative studies of parasitism at different stages in a speciation continuum [22, 23], i.e. in host populations harbouring morphs or ecotypes from no to low, moderate and high degree of genetic differentiation and reproductive isolation [24, 25]. Such approaches are important to learn more about the possible role of parasites in speciation processes. More specifically, knowledge about the when and where of infection divergence in the speciation process is important for understanding the cause and effect relationship between parasite-driven divergent selection and the build-up of reproductive isolation.

An important factor underlying variation in possible adaptive divergence among populations under parasite-mediated divergent selection is the magnitude of gene flow between the populations. Gene flow can either constrain [26–28] or facilitate adaptation [29–31], a topic which has been debated over several decades [30, 32–34]. Several different outcomes are possible in the context of parasite-mediated divergent selection and speciation. First, gene flow may constrain adaptation to spatial heterogeneity in infection conditions which may result in selection against migration, for matching habitat choice, or for assortative mating. These can all facilitate speciation and would predict either no relationship (when gene flow exceeds divergent selection) or a positive relationship (when selection exceeds gene flow) between the magnitude of infection divergence and extent of reproductive isolation. A positive relationship would also be expected when populations diverge between habitats as a result of other sources of divergent selection and subsequently become exposed to increasingly differentiated infection conditions as the specialization to contrasting habitats progresses. Second, gene flow may facilitate adaptation to spatial heterogeneity in infections, for instance if parasites evolve fast and host defence evolution is mutation-limited. In such a case, it could be expected that heterogeneous infection conditions would favour high gene flow, thus eroding reproductive isolation or restricted migration. Under such a scenario, no relationship between the infection regimes and host reproductive isolation is expected. The above scenarios would be particularly likely in sympatric or parapatric host populations where host migration and gene flow are not constrained by strong geographical barriers compared to allopatric populations that are effectively isolated by geographic barriers. To test these alternative predictions, studies are needed that explore the relationship between the magnitude of reproductive isolation and resulting neutral genetic differentiation of host populations and differences in parasite infections between them under geographical conditions that allow gene flow or make it impossible. We are aware of only one very recent study addressing this question, albeit at a large geographical scale across different continents [25]. In the present paper, we investigated macroparasite infections in replicated ecotype-pairs of parapatric lake and stream threespine stickleback (*Gasterosteus aculeatus* species complex) in Switzerland in relation to the magnitude of neutral genetic differentiation between the populations and in comparison to allopatric population contrasts.

Several freshwater fish taxa have undergone rapid adaptive radiations in northern latitudes after the end of the last glaciation [35–40]. In threespine stickleback, this has included parallel colonisation of fresh waters by marine ancestors across much of the northern hemisphere, and differentiation of freshwater populations into lake and stream ecotypes and species [41–49], and much less commonly into benthic and limnetic ecotypes [50, 51] or substrate-associated ecotypes within lakes [52]. The colonization history of the invasive threespine stickleback in the midlands of Switzerland is much younger. The range expansion started after 1870 following introductions of fish around that time, and building of irrigation channels throughout the country [48, 53]. The colonization events took place from three separate lineages: The Lake Constance lineage consists of mitochondrial haplotypes from the Baltic region, Lake Geneva is dominated by Rhone valley haplotypes, and a Swiss River Rhine lineage invaded the midlands from the Basel region north of the Jura Mountains [53]. In the course of the recent range expansion, these lineages met and formed a broad hybrid zone across northern and western parts of Switzerland resulting in high phenotypic diversity within some populations [48, 53, 54]. Currently, sticklebacks are found in diverse habitats ranging from small streams to pelagic areas of the large lakes. This sharp habitat contrast between the lake and stream ecotypes, and their parallel origin in several historically independent lineages in contemporary time [48], provide an exceptional opportunity to explore the relationship between parasite infections and the origin of reproductive isolation in young ecotype contrasts.

We focused on four lake systems in Switzerland each harbouring independently evolved parapatric pairs of lake and stream stickleback ecotypes. The Constance and Geneva systems have distinct stickleback lineages originating from drainages separated by considerable geographical distance. These lineages are also phenotypically highly distinct and belong to different nominal taxa, *G*. *aculeatus* and *G*. *gymnurus* respectively [55]. The other two lake systems, Biel and Wohlen (Bern), lie in the hybrid zone and harbour a mixture of all mitochondrial haplotypes with genetic admixture among the lineages [53]. A recent study demonstrated significant parallel differentiation in morphological (anti-predator and feeding related traits) and ecological (trophic position) traits between the lake and the stream populations among these pairs, with the extent of morphological differentiation independent of geographical distance between breeding sites, and a significant contribution of habitat difference to genetic isolation [48]. This suggests an early onset of ecological speciation in this system with some habitat-mediated reproductive isolation. Here we asked if the extent of reproductive isolation between the parapatric ecotypes, measured as neutral genetic divergence, and also the genetic divergence between allopatric populations correlated with the magnitude of parasite contrasts. We expected this relationship to be stronger in parapatric populations where gene flow was not constrained by geographical barriers.

## Materials and Methods

### Stickleback sampling and parasitological analysis

**Fig 1:**
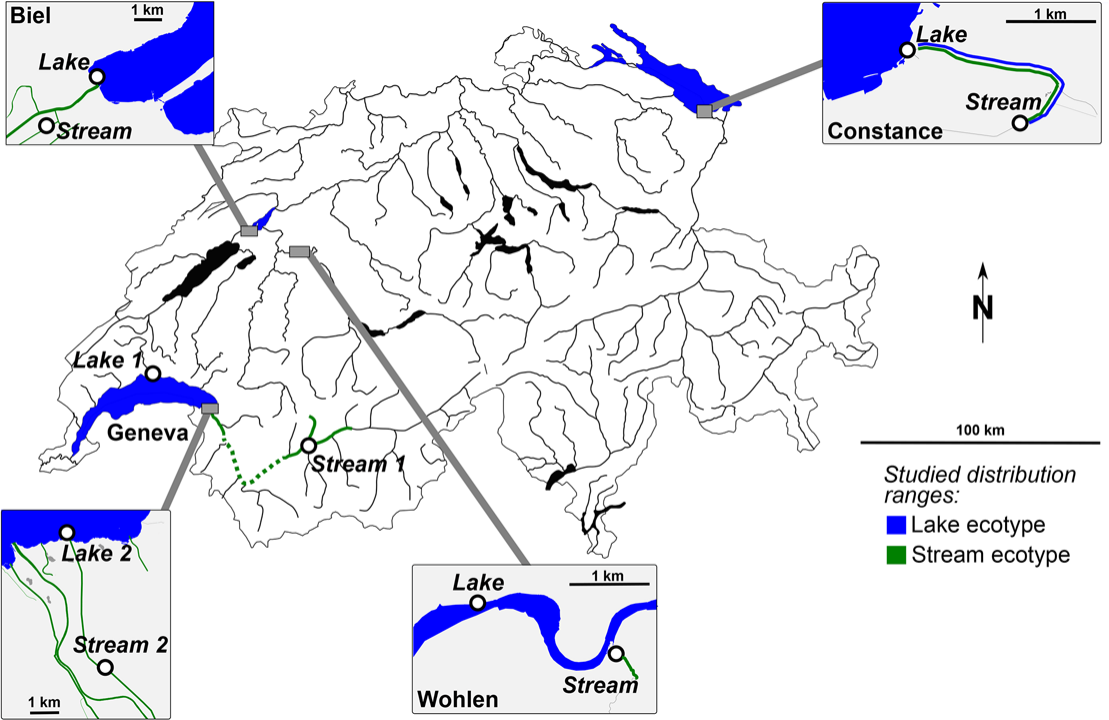
Map of the four study lake systems in Switzerland. Lake populations of stickleback are indicated in blue and stream populations (all streams are inlets flowing into the lakes) in green. The Constance and Geneva systems harbour distinct stickleback lineages while the Biel and Wohlen (Bern) systems lie in the stickleback hybrid zone. Note that the distribution ranges of lake and stream populations overlap in the Constance system as lake fish migrate upstream to spawn in spring. Map copyright: Wikimedia (CC BY-SA); Inlets copyright: OpenStreetMap contributors (CC BY-SA).

Sticklebacks of both lake and stream ecotypes were sampled from each of the four lake systems (Wohlen, Biel, Constance and Geneva, Fig 1) during the stickleback breeding season in April-May 2012 using dip nets from shallow water or un-baited minnow traps set overnight. In the Geneva system, the sampling protocol included samples from two pairs of lake and stream sticklebacks (Fig 1, S1 Table). Waterway distance between the stream sampling location and the lake shore was short in all lake systems (0.18–2.3 km), except for the locations in the Geneva system where they were longer (S1 Table). Fish were euthanized in MS-222 or clove oil solution and put on ice immediately after catching. In the laboratory, the fish were measured for standard length and dissected for macroparasite infections on gills, eyes and internal organs. Since bringing the fish alive to the laboratory was logistically not possible, but fresh material was needed for examination of eye cataracts (see below), protozoan and monogenean infections were not examined. In most cases, parasites were identified to the genus level. For example, eye flukes of the genus *Diplostomum* commonly include several co-infecting species or cryptic species that are morphologically indistinguishable [56–58]. However, *Diplostomum* species found in the eye lenses are different from those in the eye humour [57, 59] and they are referred here to as two groups of *Diplostomum* spp. according to their site of infection, lens or humour of the fish eye. The lens-infecting species of *Diplostomum* cause cataracts in eyes of fish [60]. To estimate their impact on the sticklebacks, the coverage of cataracts in the eye lenses of the fish (% of lens area) was scored from 0% to 100% in steps of 10% using slit-lamp microscopy [60].

### Microsatellite analysis

DNA was extracted for a total of 272 stickleback individuals which were in some cases caught earlier from the study sites (S2 Table). Extraction was done using 10% Chelex solution following the manufacturers protocol (Biorad, California, USA). Nine microsatellite markers were amplified in one multiplex kit. Detailed information on the marker identity, the multiplexing setup and the PCR protocol are described in [61]. Alleles were visualized on an ABI 3130XL and scored with GeneMapper 4.0 (Applied Biosystems, Switzerland). Using GenoDive 2.0 [62], deviations from Hardy-Weinberg equilibrium were calculated using 10,000 bootstrap replicates. In addition, pairwise F_ST_ [63] between parapatric and allopatric lake and stream populations were calculated for all possible population combinations. Significances were estimated using 1000 bootstrapped replicates as implemented in GenoDive.

### Statistics

Differences in the mean abundance of parasites (abundance of all parasites per fish) between the lake systems and between ecotypes within the lake systems were analysed using generalized linear models (GLM) with negative binomial probability distribution and log link function. This was done to account for the aggregated distribution of parasites, i.e. distributions skewed to the left with few host individuals harbouring a high fraction of the parasite population. Lake system, habitat and fish sex were used as fixed factors, and fish standard length as a covariate. To account for the statistical non-independency of the two lake-stream pairs from the Geneva system, the analysis was run twice including one of the pairs at a time.

To test if differences in parasite species composition and abundance within habitats across the lake systems and between habitats within and across the lake systems correlated with the extent of neutral genetic differentiation among the stickleback populations, pairwise F_ST_ calculated from variation at nine microsatellite loci were used (S2 Table). Proportional similarity in parasite species composition between all possible population combinations was calculated as Jaccard similarity: a/(a+b+c), where a is the number of parasite species found in both populations, and b and c are numbers of species found only in the first and the second host population, respectively [64]. The index was converted to dissimilarity by subtracting all values from 1. Difference in parasite abundance was calculated as an absolute difference in the mean total parasite abundance between all possible population pairs. Pairwise differences in parasite species composition and abundance were then plotted against corresponding pairwise F_ST_ values. In the Geneva system, average F_ST_ and infection differences between the two lake-stream pairs were used in the comparisons to other lake systems in parapatry (average of the four possible lake-stream combinations between Geneva Lake 1 and 2, and Stream 1 and 2 populations) and in allopatry (averages of the Geneva Lake 1 vs. Lake 2, or Geneva Stream 1 vs. Stream 2 comparisons). To account for the effect of geographical distance in the analyses, distance (direct line “as the bird flies” between two points) was determined for all population pairs. Direct distance measure was used since waterway distances in allopatric combinations would result in arbitrarily large distances because we compared populations between systems draining into different oceans while the colonisation clearly happened by crossing the water sheds at the headwaters within Switzerland [54]. However, in the parapatric pairs, we used the waterway distance between the stream sampling site and the nearest lake site as this provided a reasonable estimate of the connectivity between the populations. Again, mean distances between the locations were used in the Geneva system as described above. The relationships were analysed using Pearson correlation analysis using F_ST_ values and F_ST_ residuals from linear regression of F_ST_ against geographical distance. We do not report p-values for these correlation statistics as we only had four independent parapatric data points and in allopatry the pairwise comparisons constituted to an incomplete matrix of statistically non-independent values. However, we calculated one p-value for the complete matrix of pairwise contrasts, including the four parapatric pairs and 24 allopatric combinations, to obtain a conservative estimate of statistical significance of the correlation in allopatry. This analysis was done using partial Mantel test controlling for geographical distance with 9999 permutations of the data. All statistics were performed in IBM SPSS Statistics 20 and statistical package R version 2.15.2.

### Ethics statement

All fish of the study were euthanized in MS-222 or clove oil solution, commonly used fish anesthetics. All necessary permits to sample sticklebacks for the described field studies were obtained from the cantonal fishery authorities of cantons Bern, Vaud, St. Gallen and Valais. Fish collection followed the Swiss veterinary legislation in concordance with the federal food safety and veterinary office (FSVO) and the cantonal veterinary office in Bern (Veterinärdienst des Kantons Bern).

## Results

A total of 12 different parasite taxa were found from the 303 sticklebacks studied from the lake and stream populations (Table 1). The highest number of taxa (9) was found in the Wohlen system and the lowest number (4) in the Biel system. Eye flukes of the genus *Diplostomum* (Trematoda) transmitted to fish as free-swimming cercaria larvae from aquatic snails and glochidia larvae of bivalves (Mollusca) were among the most prevalent parasite taxa, but this depended on the host population. For example, these taxa were found in all lake populations, but they were absent from the stream populations in the Constance and Geneva systems. The acanthocephalan *Acanthocephalus lucii*, transmitted trophically to fish from isopods, was common only in the Biel system whereas the other prevalent taxa, trematode *Cyathocotyle* sp. (transmitted from snails) and acanthocephalan *Pomphorhynchus* sp. (transmitted trophically from amphipods), were found only in the Wohlen system. Lens-infecting *Diplostomum* spp. was the most abundant parasite taxon accounting for 59.4% of all parasite individuals. Coverage of cataracts caused by these parasites reached 100% of the lens area in some stickleback individuals. The highest coverages of cataracts were detected in the Wohlen system that also had the highest abundance of lens-infecting *Diplostomum* spp.

**Table 1:** Parasite infections in stickleback.

Parasite taxon	Life cycle	Wohlen		Biel		Constance		Geneva			
		Lake (n = 31)	Stream (n = 30)	Lake (n = 24)	Stream (n = 19)	Lake (n = 40)	Stream (n = 30)	Lake 1 (n = 40)	Stream 1 (n = 40)	Lake 2 (n = 25)	Stream 2 (n = 24)
*Diplostomum* spp. (Trematoda), Eye lens	Indirect (Snail)	90.3 (5.1±0.7)	90.0 (5.2±0.8)	95.8 (3.9±0.6)	100.0 (4.0±0.5)	30.0 (0.6±0.1)	0	80.0 (2.4±0.4)	0	44.0 (0.8±0.2)	0
*Diplostomum* spp. (Trematoda), Eye humour	Indirect (Snail)	48.4 (1.1±0.3)	30.0 (0.6±0.3)	4.2 (0.1±0.1)	0	2.5 (0.02±0.02)	0	2.5 (0.02±0.03)	0	24.0 (0.3±0.1)	0
*Apatemon* sp. (Trematoda), Eye humour	Indirect (Snail)	0	0	0	0	0	0	0	0	44.0 (0.6±0.2)	0
*Cyathocotyle* sp. (Trematoda), Gills/body cavity	Indirect (Snail)	58.1 (2.8±1.1)	60.0 (2.1±0.6)	0	0	0	0	0	0	0	0
*Proteocephalus* sp. (Cestoda), Intestine	Indirect (Copepod)	0	0	0	0	5.0 (0.05±0.03)	3.3 (0.03±0.03)	0	2.5 (0.03±0.03)	0	4.2 (0.04±0.04)
*Schistocephalus* sp. (Cestoda), Body cavity	Indirect (Copepod)	3.2 (0.03±0.03)	0	0	0	5.0 (0.05±0.04)	0	0	0	0	0
*Raphidascaris acus* (Nematoda), Body cavity	Indirect (Aquatic invertebrates)	0	0	0	0	2.5 (0.02±0.03)	0	0	0	0	0
Unidentified nematode (Nematoda), Intestine	Indirect	0	3.3 (0.03±0.03)	0	0	0	0	0	0	0	0
*Acanthocephalus lucii* (Acanthocephala), Intestine	Indirect (Isopod)	0	13.3 (0.3±0.2)	45.8 (0.7±0.2)	63.2 (1.0±0.2)	0	0	0	5.0 (0.05±0.03)	16.0 (0.3±0.1)	0
*Echinorhynchus* sp. (Acanthocephala), Intestine	Indirect (Amphipod)	0	6.7 (0.07±0.05)	0	0	0	0	0	0	0	0
*Pomphorhynchus* sp. (Acanthocephala), Intestine	Indirect (Amphipod)	29.0 (0.5±0.2)	33.3 (0.8±0.3)	0	0	0	0	0	0	0	0
Glochidia (Mollusca), Gills	Direct	3.2 (0.03±0.03)	13.3 (0.2±0.1)	4.2 (0.1±0.1)	21.1 (0.3±0.1)	40.0 (1.3±0.4)	0	30.0 (0.6±0.2)	0	4.0 (0.08±0.08)	0

Prevalence (% fish infected), mean abundance (mean number of parasites per fish ± SE), and the site of infection of the 12 macroparasite taxa observed in lake and stream ecotypes of threespine stickleback from four lake systems (Wohlen, Biel, Constance and Geneva) in Switzerland. ‘Life cycle’ indicates whether the parasite life cycle includes only the fish host (direct life cycle) or also other host taxa (indirect life cycle). Previous host in the life cycle is given in parentheses.

All genetic markers were in Hardy-Weinberg equilibrium (all *p* > 0.05, results not shown). Parapatric lake and stream stickleback populations differed at neutral genetic markers in the Constance (*F*_ST_ = 0.038, *p* = 0.001) and in one of the two Geneva pairs (Lake 1 vs Stream 1: *F*_ST_ = 0.053, *p* = 0.001), but not in the Wohlen (*F*_ST_ = -0.005, *p* = 0.699) and Biel systems (*F*_ST_ = 0.011, *p* = 0.129) nor in the second Geneva pair (Lake 2 vs. Stream 2: *F*_ST_ = -0.003, *p* = 0.593; S2 Table). Allopatric populations differed significantly except for the populations from the Wohlen and Biel systems and to some degree in the Lake Geneva system, which is consistent with previous findings [48, 54].

**Fig 2:**
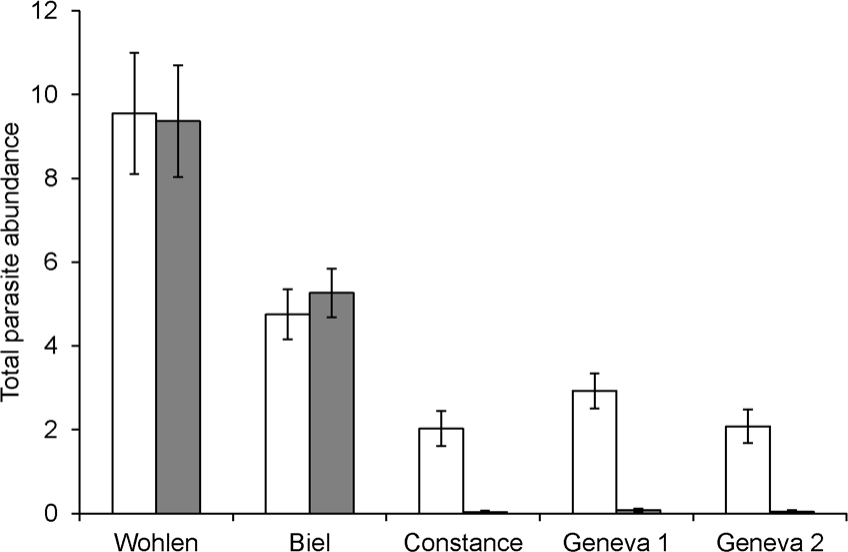
Parasite abundance differs between lake and stream ecotypes. Mean total parasite abundance (±SE) in lake (open bars) and stream (grey bars) ecotypes of threespine stickleback in four lake systems (Wohlen, Biel, Constance, Geneva) in Switzerland.

Total parasite abundance was significantly different among the lake systems (GLM: Wald = 114.18, df = 3, p < 0.001) so that the Wohlen and Biel systems had the highest parasite abundances (Fig 2). The lake populations had higher overall parasite abundance compared to their parapatric stream populations (Wald = 31.81, df = 1, p < 0.001), while there was also a significant interaction between the factors lake system and habitat (Wald = 42.73, df = 3, p < 0.001). This was caused by the higher parasite abundance in the lake populations in the Constance and Geneva systems but even abundances in lake and stream populations in Biel and Wohlen. Fish length as a covariate had a marginally significant positive effect (Wald = 2.95, df = 1, p = 0.086). The main effect of fish sex (p = 0.227), or its interactions with the other factors (p > 0.6 for all), were not significant indicating that males and females had similar parasite abundances within the lake systems and between the ecotypes. As a consequence, sex was not included in the final GLM. Using data from the other lake-stream pair in the Geneva system did not change these results.

**Fig 3:**
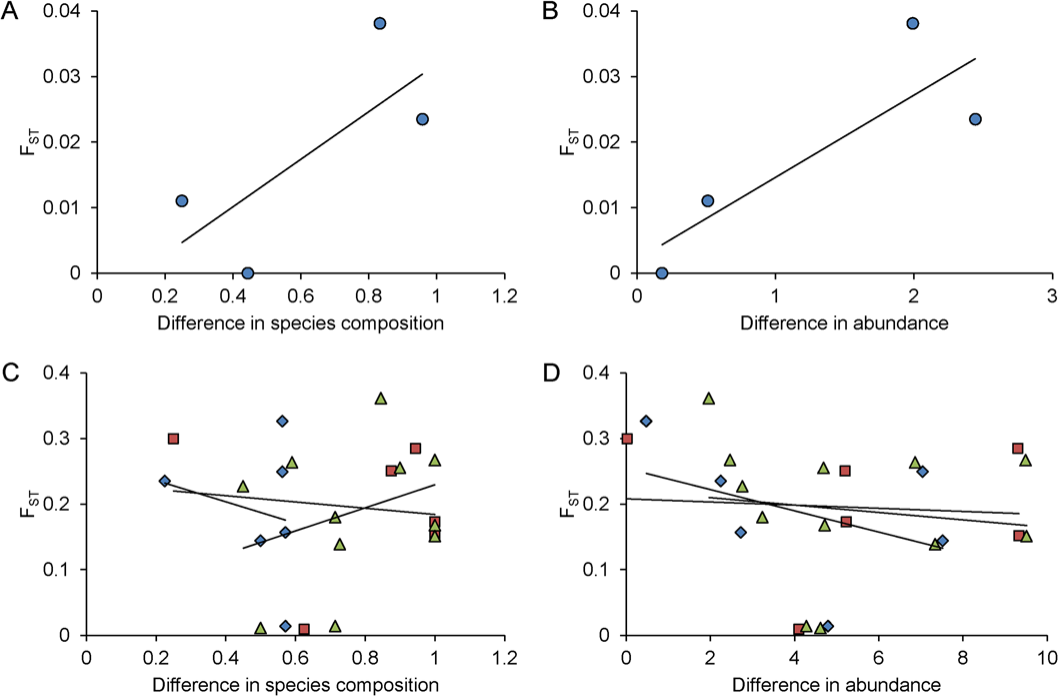
Genetic differentiation tends to correlate with infection difference in parapatry. Magnitude of genetic differentiation, F_ST_ and F_ST_ residual from geographic distance, in relation to proportional difference in parasite species composition and difference in parasite abundance in parapatric lake-stream pairs (panels A and B) and in allopatric population combinations (panels C and D) of threespine stickleback (*Gasterosteus aculeatus*) from four lake systems (Wohlen, Biel, Constance and Geneva) in Switzerland. Symbols refer to different population combinations: parapatric lake-stream pairs (dots); allopatric lake-lake pairs (diamonds), stream-stream pairs (squares) and lake-stream pairs (triangles). Fitted lines represent linear regressions to indicate the slope and intercept of the relationships.

Correlations between the magnitude of differentiation at neutral genetic markers, and difference in parasite species composition and parasite abundance between parapatric lake and stream populations, were strongly positive (Pearson correlation: r = 0.731 and r = 0.844, respectively), indicating that parasite infections tended to become more strongly differentiated when stickleback ecotypes were more strongly differentiated genetically (Fig 3). These correlations remained positive after controlling for the effect of geographical distance between the sampling locations on F_ST_ (Table 2). This pattern was mainly caused by variation among systems in the differences of lens-infecting *Diplostomum* spp. abundance between lake and stream stickleback (Pearson correlation only for the difference in abundance of *Diplostomum* spp. in the parapatric pairs: r = 0.490). Difference in prevalence of the lens-infecting *Diplostomum* spp. tended to correlate with the magnitude of genetic differentiation too (r = 0.638).

**Table 2:** Correlations in allopatric and parapatric population combinations.

	F_ST_		F_ST_ residual from distance	
	Species composition	Abundance	Species composition	Abundance
Lake-Lake	r = -0.21	r = -0.42	r = -0.43	r = -0.25
Stream-Stream	r = -0.13	r = -0.08	r = -0.04	r = 0.07
Lake-Stream	r = 0.34	r = -0.14	r = 0.07	r = -0.04
Parapatric Lake-Stream	r = 0.73	r = 0.84	r = 0.57	r = 0.68

Pearson correlations between differences in parasite species composition and parasite abundance, and the magnitude of genetic differentiation F_ST_ and F_ST_ residual from geographical distance, in allopatric and parapatric population comparisons of lake and stream ecotypes of threespine stickleback.

Correlations between pairwise F_ST_ and pairwise difference in parasite species composition and abundance in allopatric lake-lake, stream-stream, and lake-stream population combinations were generally weak and inconsistent in direction (Table 2, Fig 3), indicating that the magnitude of genetic differentiation between allopatric stickleback populations was unrelated to the magnitude of parasite infection contrasts. This was supported also by the correlation analysis on the whole matrix of pairwise contrasts (including the four parapatric and the 24 allopatric contrasts, mean values across the populations in the Geneva system) between F_ST_ and infection divergence (partial Mantel test controlling for geographical distance: r = -0.185, p = 0.753 (difference in parasite species composition); r = 0.129, p = 0.199 (difference in parasite abundance)).

## Discussion

The role of parasite-mediated divergent selection in ecological speciation and adaptive radiations of freshwater fishes has attracted considerable attention in recent years [10]. However, examinations of parasitism in continuums of host genetic differentiation, i.e. speciation continuums [22, 23], are lacking although such systems allow questions related to the role of parasites in the very early stages of a speciation process [24, 25]. By examining parasite infections in replicated pairs of geologically very young parapatric lake and stream threespine stickleback ecotypes in Switzerland, we explored the relationship between the magnitude of recent or current gene flow and distance in parasite infections in comparison to allopatric population pairs between different drainage systems. We found that the extent of neutral genetic differentiation tended to be positively correlated with the differences both in parasite species composition and parasite abundance among the parapatric stream-lake contrasts, so that infections became more differentiated with increasing genetic differentiation. However, no such relationship was observed in contrasts among geographically isolated lake and stream stickleback populations. This suggests that, while the magnitude of infection contrasts is unrelated to neutral genetic differentiation between stickleback populations when gene flow is constrained by geographical barriers, in parapatry, where gene flow is possible, infection divergence and neutral genetic differentiation are related to each other. However, we also observed that one of our two ecotype pairs from the Geneva system showed differentiation in infections in the absence of neutral genetic differentiation. While this suggests variation in the relationship between genetic differentiation and differences in infections among the population pairs even within one lake system, the result is consistent with the idea that differentiation in infections is present as soon as two populations occupy different environments, in which case it precedes the differentiation in neutral markers. In other words, our data suggest that infection divergence could be important at early onset of a speciation process. However, many more replicated population contrasts are needed to address the generality of our findings and experimental work is needed to test hypothesis regarding causality.

In general, variation in parasite infections arises as a consequence of differences in exposure and/or susceptibility between host individuals and populations [65, 66]. In the present study, stream sticklebacks had significantly fewer infections compared to lake sticklebacks in three of the populations, which is in accordance with previous studies on lake-stream pairs of threespine stickleback [25, 67–69]. For example, Scharsack et al. [68] reported no infections from trematodes in river and lake sticklebacks caged in a river, while both river and lake sticklebacks became readily infected with trematodes in a lake exposure. We found a similar result in the Constance and Geneva stream populations, where the fish had neither trematode infections nor glochidia larvae of bivalves. These differences are likely to be explained for a large part by lower levels of exposure in streams and rivers due to absence, low abundance or low infection prevalence of certain intermediate host taxa. However, Lymnaeid snails, first intermediate hosts for trematodes such as *Diplostomum* spp., are nevertheless known to occur in these water systems (Swiss faunal center: http://lepus.unine.ch). Similarly, the differences in infections of trophically transmitted parasites such as cestodes and acanthocephalans may reflect the previously shown dietary differences between the ecotypes in this system ([48, 70], Lucek unpublished data). However, overall these differences in infections were weaker and more inconsistent compared to trematode infections.

Many parasite taxa, such as larval trematodes, can also live in fish for several years serving as a reservoir for the parasite population, which can provide information of the movements of the fish over long periods of time. For example, while details of the movements and migrations of these fishes within a year are largely unknown, absence of trematode infections in stream sticklebacks in the Constance and Geneva systems suggests that, despite in some cases only two kilometres of physical distance, these fish do not migrate to the lakes, at least not during summer months when the infections in the lakes are likely to take place. In contrast, occurrence of these parasites in stream stickleback of the Wohlen and Biel systems suggests that the stream sticklebacks there do visit the lake habitats to a certain extent. Since these infection patterns tended to be correlated with the magnitude of genetic differentiation between the ecotypes, our results suggest that neutral marker differentiation among populations that are not geographically isolated is a good proxy for the degree of habitat specialization in this system. On the other hand, differences in infections between ecotypes in the absence of neutral genetic differentiation in one of our population pairs from the Geneva system could imply, for instance, recent establishment of the populations such that they are not yet distinguishable in neural genetic markers.

Consistent habitat segregation and habitat-specific infection may favour parasite-mediated divergent selection already at early stages of population differentiation. A recent study on this system has demonstrated that adaptive divergence in morphological traits associated with defence and feeding in these same lake-stream pairs significantly supersedes that expected from the magnitude of neutral genetic differentiation. In other words, strong adaptive differentiation has taken place independently in parallel between the lake and stream populations of stickleback in several lake systems. This previous study also revealed that phenotypic differentiation of ecotypes was a better predictor of neutral gene flow than geographical distance, suggesting that divergent adaptation is limiting gene flow between some of the ecotype populations, thus signalling the very early stages of ecological speciation [48]. The observed differences in parasite pressure could contribute to population differentiation, e.g. through immunological adaptation of fishes to specific infection conditions. Habitat-specific adaptation in parasite resistance and immune responses has been described in lake and river populations of stickleback in Germany [68, 71]. Moreover, local adaptation in major histocompatibility genes associated with mate choice [8, 72] has been documented in hybridization experiments between lake and river stickleback populations [69]. However, the state of immunological adaptation in the Swiss stickleback populations is currently unknown. It should also be noted that we sampled these populations at one time point, which does not allow analyses on the temporal consistency of the infection differences. Recent studies on spatiotemporal patterns of parasitism in sticklebacks, however, support at least short-term consistency in macroparasite infections in replicated population samples [73], while longer-term data are clearly needed to address this question.

One of the prerequisites of parasite-mediated divergent selection is that infections that differ between populations have fitness consequences on the hosts. In general, estimating the effects of parasitism on host fitness in natural populations is challenging and empirical data are scarce [17]. Moreover, as hosts typically are infected with multiple interacting parasite taxa at the same time, effects and selection caused by individual parasites are difficult to separate [10]. Certain parasite taxa infecting the stickleback in this system are nevertheless known to be potentially harmful. Clearly the most prevalent, abundant and widespread parasite taxon was *Diplostomum* spp., trematodes that infect eye lenses of fish. These parasites cause cataracts which are associated with impairment of vision, and reduced feeding efficiency and growth [74, 75], as well as increased susceptibility to predation [76]. We found that cataracts with a high coverage of the lens area may develop already at low parasite abundances, which is in accordance with earlier results of stickleback being especially sensitive to deleterious effects of the infection [77]. This is probably because of the small size of the eye lens in stickleback in relation to the size of the parasites when only few parasite individuals may be required to cover large areas of the lens area and obscure or obstruct the vision of the fish. Interestingly, we found that the magnitude of neutral genetic differentiation between the parapatric lake and stream populations tended to be positively correlated with differences in prevalence and abundance of the lens-infecting *Diplostomum* spp. parasites. This suggests parasite-taxon consistency in the infection divergence across several parapatric ecotype pairs. *Diplostomum* parasites are also associated with infection differences between sympatric limnetic and benthic stickleback species in Canada [17], sticklebacks living in lava rock and mud habitat substrates in Iceland [19, 20], and in parapatric lake and river or stream ecotypes of stickleback in Germany [68] and Switzerland (this study). In the two latter cases that studied similar habitat contrasts, directions of infection differences are parallel, i.e. lower *Diplostomum* spp. infection in river and stream populations. In the German populations, higher *Diplostomum* infestation in the lake stickleback has also resulted in higher resistance of these fish against the parasite [71]. Overall, this suggests some degree of parasite taxon-specific consistency in infections of stickleback ecotypes also over larger geographical scales.

In addition to *Diplostomum*, also other parasite taxa can have negative effects on the fish. For example, *Schistocephalus* cestodes (found occasionally in two of the populations) and *Gyrodactylus* monogeneans (not studied here) are known to be associated with fitness reduction and adaptation in resistance genetics in stickleback [69, 78, 79]. Recent experimental trials with threespine stickleback have shown that differences in infection from different parasite species (in this case two nematode species) between experimental host populations can lead to rapid changes in allele frequencies at genes of the immune system [80]. This suggests that parasites can drive immunological adaptation in stickleback, while in natural populations this effect can be obscured by co-infections from multiple parasite taxa with heterogeneous effects and selection on the hosts.

While the magnitude of differences in parasite infections tended to be positively correlated with the magnitude of neutral genetic differentiation in the parapatric host population pairs that we studied, there was no such relationship among allopatric stickleback populations, no matter whether they occupied different or similar habitats. This was expected because parasite-mediated divergent selection is not expected to act on alleles at neutral microsatellite loci and because variable F_ST_ values between populations occupying the different drainage systems in Switzerland reflect variable colonisation times and histories (variable contributions of several different invasive lineages) rather than variable extents of divergent adaptation and reproductive isolation [53]. The four drainage systems nevertheless shared, on average, 46% of the parasite taxa (range 36–67%), which indicates that relatively high similarity in parasite species composition can be maintained in allopatric stickleback populations over long geographical and evolutionary distances between the populations. In the present study, a large part of the similarity in parasite species composition between lake populations in different drainage systems came from trematodes disseminated by avian definitive hosts through which parasite dispersal is less sensitive to geographical distance [81, 82].

To conclude, our results are consistent with other data that suggest that the magnitude of gene flow between parapatric populations of stickleback occupying contrasting habitats is mediated by variation in the extent of specialization and adaptation to lake or stream habitat. This would also mediate variation in the extent of differences in parasite infection, probably reflecting differences in parasite exposure. This pattern was evident both in terms of parasite species composition and infection abundance. Our data thus demonstrate potential for parasite-mediated divergent selection between populations occupying contrasting habitats already at the incipient stages of differentiation, while habitat-specific divergent adaption of the ecotypes to parasite infections remains to be demonstrated experimentally. On the other hand, variation in the extent of genetic differentiation between population pairs with similar magnitude of infection divergence from the same lake system emphasizes that data on many more ecotype pairs is needed to address the generality of our findings. Our results also imply that relatively high similarity of the parasite fauna can be maintained across distant populations owing to a common ‘core’ of parasite taxa. This suggests that it may be possible to detect parallel infection divergence and possibly parallel adaptation in pairs occupying similar habitat contrasts at large geographical scales.

## Supporting Information

S1 Original Data (XLSX)

S1 Table Sampling protocol. (PDF)

S2 Table Pairwise F_ST_. (PDF)
