# Supplementary material for: Divergent Macroparasite Infections in Parapatric Swiss Lake-Stream Pairs of Threespine Stickleback (Gasterosteus aculeatus)
Source: PLoS One. 2015 Jun 18;10(6):e0130579. doi: 10.1371/journal.pone.0130579 (PMC4472517; doi:10.1371/journal.pone.0130579)
Supplement: S1 Table — (PDF) [file pone.0130579.s002.pdf]

Table S1. Sampling protocol. Stickleback sampling dates, coordinates of the sampling locations, and waterway distance to the lake from the stream sampling site in the four study lake systems (Wohlen, Biel, Constance and Geneva) in Switzerland.

**Lake system**	**Population**	**N of fish**	**Date**	**N**	**E**	**Distance**
Wohlen	Lake	31	8.5.2012	46°57'59"	7°21'09"	
	Stream	30	8.5.2012	46°57'43"	7°22'47"	0.2 km
Biel	Lake	24	8.5.2012	47°03'05"	7°04'33"	
	Stream	19	18.5.2012	47°02'24"	7°03'29"	2.1 km
Constance	Lake	40	5.4.2012	47°29'09"	9°32'39"	
	Stream	30	5.4.2012	47°28'43"	9°33'30"	2.3 km
Geneva	Lake 1	40	24.4.2012	46°31'02"	6°34'41"	
	Stream 1	40	23.4.2012	46°12'51"	7°18'53"	61.0 km
Geneva	Lake 2	25	7.5.2012	46°23'45"	6°53'18"	
	Stream 2	24	30.4.2012	46°20'52"	6°54'38"	6.5 km
