# Supplementary material for: Divergent Macroparasite Infections in Parapatric Swiss Lake-Stream Pairs of Threespine Stickleback (Gasterosteus aculeatus)
Source: PLoS One. 2015 Jun 18;10(6):e0130579. doi: 10.1371/journal.pone.0130579 (PMC4472517; doi:10.1371/journal.pone.0130579)
Supplement: S2 Table — (PDF) [file pone.0130579.s003.pdf]

Table S2: Pairwise F_{ST}. Pairwise genetic differentiation among the lake and stream stickleback ecotypes from four lake systems in Switzerland estimated as F_{ST} (below diagonal) and respective *p* values (above diagonal) based on a bootstrap procedure with 1000 replicates. The sample sizes (n) and sampling year for each population are indicated. See main text for details.

**System**			Wohlen		Biel		Constance		Geneva			
	**Habitat**	**Year**	Lake	Stream	Lake	Stream	Lake	Stream	Lake 1	Stream 1	Lake 2	Stream 2
Wohlen	Lake (n=30)	2007	-	0.722	0.027	0.074	0.001	0.001	0.001	0.001	0.001	0.001
	Stream (n=19)	2012	-0.005	-	0.105	0.209	0.001	0.001	0.001	0.001	0.001	0.001
Biel	Lake (n=28)	2012	0.014	0.011	-	0.158	0.001	0.001	0.001	0.001	0.001	0.001
	Stream (n=13)	2012	0.014	0.009	0.011	-	0.001	0.001	0.001	0.001	0.001	0.001
Constance	Lake (n=30)	2007	0.144	0.139	0.157	0.180	-	0.001	0.001	0.001	0.001	0.001
	Stream (n=32)	2012	0.151	0.152	0.168	0.173	0.038	-	0.001	0.001	0.001	0.001
Geneva	Lake 1 (n=30)	2008	0.234	0.248	0.219	0.208	0.303	0.246	-	0.001	0.163	0.100
	Stream 1 (n=30)	2007	0.238	0.258	0.229	0.226	0.343	0.284	0.053	-	0.001	0.001
	Lake 2 (n=30)	2012	0.264	0.279	0.252	0.246	0.349	0.288	0.007	0.031	-	0.593
	Stream 2 (n=30)	2012	0.296	0.311	0.281	0.275	0.379	0.315	0.010	0.065	-0.003	-
